# Spectral domain optical coherence tomography in patients after successful management of postoperative endophthalmitis following cataract surgery by pars plana vitrectomy

**DOI:** 10.1186/1471-2415-14-76

**Published:** 2014-06-02

**Authors:** Otto Alexander Maneschg, Éva Volek, János Németh, Gábor Márk Somfai, Zsuzsanna Géhl, Irén Szalai, Miklós Dénes Resch

**Affiliations:** 1 Department of Ophthalmology, Semmelweis University, Budapest, Hungary

**Keywords:** Spectral domain optical coherence tomography, Postoperative endophthalmitis, Enhanced depth imaging, Choroidal thickness, Vitrectomy

## Abstract

**Background:**

Acute severe postoperative endophthalmitis may lead to severe vision loss. The aim of this study was the analysis of macular microstructure imaged by spectral domain optical coherence tomography in patients after pars plana vitrectomy due to postcataract endophthalmitis.

**Methods:**

A cross sectional study was carried out in 17 patients who had cataract surgery in both eyes and underwent unilateral pars plana vitrectomy due to postcataract endophthalmitis. Postoperative best corrected visual acuity was determined in both eyes. Evaluation of macular thickness, macular volume, peripapillary retinal nerve fiber layer thickness and choroidal thickness using enhanced depth imaging technique was performed by spectral domain optical coherence tomography. The measurements obtained in the operated eye were compared to the fellow eye by Wilcoxon matched pair test. Correlation test was performed by Spearman rank order.

**Results:**

A mean postoperative best corrected visual acuity of 63 ± 30 ETDRS letters versus 75 ± 21 letters was achieved in the study and fellow eyes, respectively, after a mean of 5.3 ± 4.5 months (p = 0.1). The mean macular thickness was 320.6 ± 28.8 μm SD in the study eyes compared to 318.4 ± 18.8 μm in the fellow eyes (p = 0.767). No differences were noted in macular volume (p = 0.97) and in peripapillary retinal nerve fiber layer thickness (p = 0.31). Choroidal thickness was significantly lower in the study eyes compared to the fellow eyes (p = 0.018). Epiretinal membrane was found in 7 eyes after endophthalmitis, while in the fellow eyes only in 3 cases (p = 0.13, Fisher’s exact test).

**Conclusion:**

Choroidal thickness decreased significantly after endophthalmitis, but there was no functional correlation with the changes in choroidal microstructure. The development of epiretinal membranes may be associated with either vitrectomy or endophthalmitis in the history. Absence of other significant structural and morphological findings shows that successful treatment may guarantee good clinical results even in long term after this severe postoperative complication.

## Background

Postoperative endophthalmitis is one of the most severe complications after successful cataract surgery. Improvement of pre- and postoperative hygienic and therapeutic treatments reduced the risk of development of this complication. According to recent data, the prevalence of postcataract endophthalmitis is around 0.058% in Hungary [1]. For acute severe postoperative endophthalmitis, early vitrectomy is fundamental for the treatment, especially in cases with poor initial visual acuity [2,3]. A number of factors are known to influence clinical outcomes after successful management of postcataract endophthalmitis but there are no specific data about the microstructural changes in the retina and the choroid long time after this severe postoperative complication.

It is known that a functionally normal choroidal morphology is essential for retinal function as abnormal choroidal vasculature and blood flow can result in dysfunction and death of photoreceptors [4]. Changes in choroidal thickness seem to play an exceptionally important role in the pathophysiology of many diseases, such as central serous chorioretinopathy [5], age-related macular degeneration [6,7], Vogt-Koyanagi-Harada disease [8] and other pathologies.

Optical coherence tomography (OCT) revolutionized the understanding and treatment of macular diseases. The higher acquisition speed of spectral domain OCT (SD-OCT) minimizes motion artefacts and allows a higher resolution of retinal structures [9], thus providing more extensive morphological details [10]. In recent studies, SD-OCT technology was shown to have a high accuracy and reproducibility in the imaging of retinal structures, retinal nerve fiber layer (RNFL), choroidal and corneal thickness measurements [11-15]. Many authors using enhanced depth imaging (EDI)-OCT reported satisfactory examination options and measurements of choroidal pathologies which promise choroidal OCT imaging to become a standard diagnostic procedure [5,16].

The advantage of OCT imaging is its non invasive nature with minimal risk for the patients. In addition, the presence of structural retinal and choroidal changes due to the severe complications of endophthalmitis may help to predict the outcomes after vitrectomy. Therfore, the main goal of this study was to analyze the retinal and choroidal microstructure imaged by SD-OCT in patients after pars plana vitrectomy due to postcataract endophthalmitis.

## Methods

A cross sectional, observational study was carried out between 1 July 2012 and 31 January 2013 at the Department of Ophthalmology, Semmelweis University, Budapest, Hungary. The enrolled patients had undergone bilateral cataract surgery and PCL implantation with postoperative endophthalmitis in one eye. Our department provides the regional tertiary care for endophthalmitis and therefore the majority of postcataract endophthalmitis cases are referrals from surgical centers performing the surgeries. The study was approved by the Ethical Committee of Semmelweis University, Budapest and the Hungarian Human Subjects Research Committee. All patients provided written informed consent. The study was conducted according to the tenets of the Declaration of Helsinki.

Patient charts were evaluated retrospectively where pars plana vitrectomy was performed in the period between 2008 and 2012 due to severe acute endophthalmitis following cataract surgery and obtained clear optic media after recovery. Twenty-five patients were invited to participate in the study, seventeen patients agreed to visit our department and give consent. The age range was 56 to 89 years (69.5 ± 7.8 years, mean ± SD), 7 patients were female. All patients underwent phacoemulsification and posterior chamber intraocular lens implantation in both eyes. The patients developed postoperative endophthalmitis between 2008 and 2012. The acute onset postoperative endophthalmitis cases – all within 8 days after successful cataract surgery – were managed by pars plana vitrectomy (with complete detachment of the posterior hyaloid confirmed by intraoperative triamcinolone staining) performed within 24 hours of the outbreak. Within 4 weeks after vitrectomy, all patients reached clear optical media. The average time for the SD-OCT assessment performed after the vitrectomy was 48 ± 34 months.

Only patients with artificial intraocular lens bilaterally were enrolled to reach similar postoperative conditions. Exclusion criteria included known ocular diseases such as glaucoma, diabetic retinopathy or exudative age-related macular degeneration (AREDS 3 classification or higher). Patients with high myopia, over minus 6 dioptres or with an axial lengh over 26 mm were also excluded from the study. Two patients were myopic with an axial lengh under 26 mm.

First, the refractive power was determined with an autorefractor keratometer and BCVA (best corrected visual acuity) was assessed by using ETDRS charts in both eyes of all patients. Then slit-lamp examination of the anterior segment was performed followed by fundoscopic examination after pupillary dilation. SD-OCT examinations were performed in all eyes by a single experienced examiner (EV) using Spectralis (Heidelberg Engineering, Heidelberg, Germany) SD-OCT, which provides up to 40000 A-scans per second with 7 μm depth resolution in tissues and 14 μm transversal resolution of images of ocular microstructures. Correct posture, head position, focus on the video imaging and centralization of the scan area were carefully monitored along with optimal scan settings. After each examination, the best image was assessed. Using the standard software of Spectralis OCT (Spectralis software v.5.1.1.0; Eye Explorer Software 1.6.1.0, Heidelberg Engineering), we assessed the central and peripheral macular thickness and macular volume. The presence of epiretinal membrane was recorded in both groups along with the presence of severe traction (i.e. traction causing disappearance of the foveal contour). Peripapillary retinal nerve fiber layer (RNFL) thickness measurements were performed using a 12-degree diameter circular scan pattern. The average RNFL thickness value provided by the software was used for further analyses.

**Figure 1:**
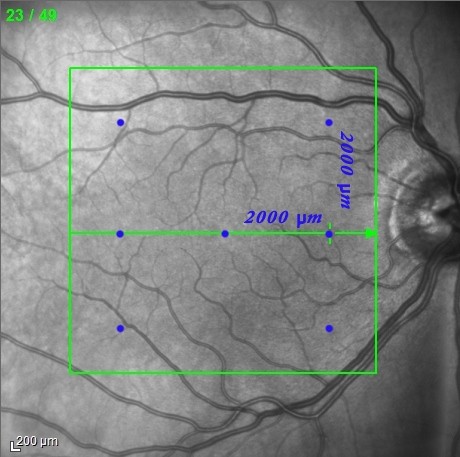
**The blue dots on the infrared fundus image denote the measurement points used in the study.** Each measurement point has a distance of 2000 um on the central horizontal and two vertical axes.

**Figure 2:**
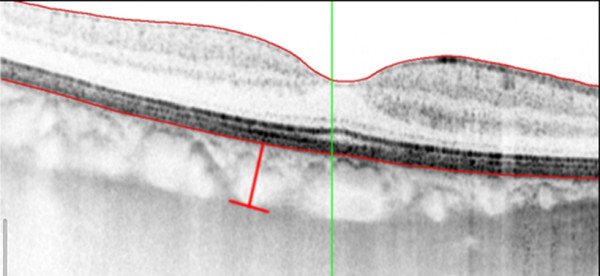
**SD-OCT image in EDI mode in an eye after postoperative endophthalmitis.** Choroidal thickness is measured between the outer border of the retinal pigment epithelium and the inner scleral border using the caliper tool of the software (red line).

For the measurement of choroidal thickness patients underwent enhanced depth imaging spectral-domain optical coherence tomography which was obtained by positioning the device close to the eye and employing the automatic EDI mode of the device. A horizontal linear section comprising 50 averaged scans was obtained of each macula within a 20° × 20° area. The OCT protocol was performed focusing on the fovea. Choroidal thickness was measured in 7 manually selected points in the macula by using a caliper scale provided by the software of the SD-OCT device: one in the fovea, two points located temporally and nasally from the fovea in the horizontal meridian at a distance of 2000 μm, and four points located superior and inferior to the temporal and nasal horizontal measurement locations, also at a distance of 2000 μm (Figure 1). Choroidal thickness was measured by the caliper tool from the outer border of the retinal pigment epithelium to the inner scleral border (Figure 2). All measurements were conducted by a second independent examiner (OM) who was masked to the patient and eye data that were analyzed.

Pairwise comparisons were made between the post-endophthalmitis eye (study eye) and the fellow healthy eye (control eye). The statistical analyses were done using the Statistica 8.0 software (Statsoft Inc., Tulsa, USA). Data were expressed as mean values ± standard deviation. Wilcoxon nonparametric test was used for the comparison of thickness data between the study and control eyes. The occurrence of epiretinal membranes was compared by Fisher exact test. Spearman rank order correlation test was performed between central retinal thickness and subfoveal choroidal thickness. The level of significance was set at p < 0.05.

## Results

**Figure 3:**
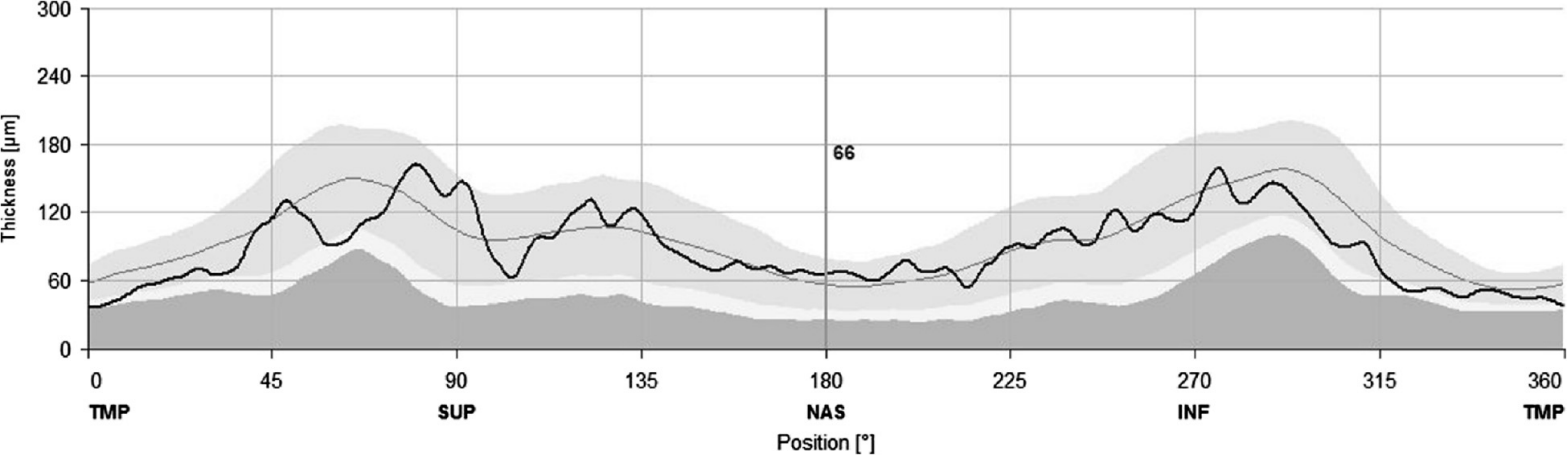
**Measurement of the peripapillary nerve fiber layer thickness in an eye after postcataract endophthalmitis.** Note that the thickness curve is running mostly within normal limits, except for the temporal and superotemporal regions.

The mean visual acuity of the patiens before performing vitrectomy was 0.03, 11 of them had a visual acuity of HM (hand movement) and 2 subjects had only LP (light perception). The patients were treated intraoperatively and after vitrectomy with vancomycin/amikacin, ceftazidim and steroids for an average period of 8 days. Vitrectomy was performed in all cases without complication, there were no vitreous hemorrages or retinal detachments during or after the surgeries. Microorganisms were isolated from eight specimens with seven cases of staphylococcus spp. among them. The mean postoperative BCVA was 63 ± 30 ETDRS letters in the study eye group and 75 ± 21 ETDRS letters in the control group (p = 0.1). The mean retinal thickness in the study eyes was 320.6 ± 28.83 μm and 318.4 ± 18.8 μm in the control eye group (p = 0.767) and there was no difference in thickness of the remaining eight macular regions, either. (Table 1) The endophthalmitis group showed a mean macular volume of 8.79 ± 0.92 μm^3^ and 8.9 ± 0.91 μm^3^ in the control eyes (p = 0.97). In the endophthalmitis study eye group, the mean RNFL thickness was 92.2 ± 15.1 μm, while it was 97.8 ± 18.4 μm in the control eye group, the difference was not significant (p = 0.31). In 4 cases of the endophthalmitis eyes, the software assessed the peripapillary mean RNFL thickness being below normal or borderline, compared to 3 RNFL measurements in the control eyes. (Figure 3).

**Table 1:** Retinal thickness changes in the different macular regions in the study groups (mean ± SD)

**Macular region**	**Endophthalmitis (study ) eye in μm**	**Control (fellow) eye in μm**	**p value**
sup. near	303 ± 51.56	308.9 ± 40.69	**0.68**
sup. far	358.6 ± 44.52	335.7 ± 46.08	**0.27**
nas. near	306.9 ± 37.63	314.3 ± 25.06	**0.68**
nas. far	359.3 ± 46.94	344.9 ± 54.5	**0.68**
inf. near	297.9 ± 57.85	295.3 ± 34.95	**0.61**
inf. far	348.6 ± 43.45	335.9 ± 47.46	**0.2**
temp. near	279.4 ± 44.38	297.4 ± 50.14	**0.97**
temp. far	325.3 ± 49.2	331.5 ± 43.02	**0.91**
central (CRT)	306.7 ± 78.35	302 ± 82.17	**0.66**
**Mean ± SD**	320.6 ± 28.83	318.4 ± 18.8	**0.76**

In six eyes of four patients, early stages of age related macular degeneration (stage 1–2 AREDS classification) was detected with slight pigment alteration and drusen but no lesion activity.

Other frequent clinical findings in the study group was the development of epiretinal membranes (7 cases vs. 3 cases in the fellow eyes, p = 0.13, Fisher exact test), all without severe traction.

**Figure 4:**
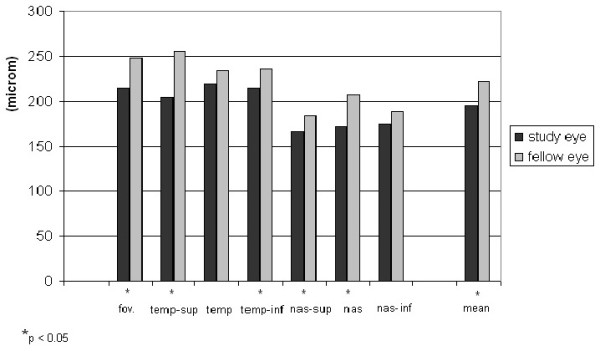
**Choroidal thickness in the different measurement regions and mean choroidal thickness comparison between study eye and fellow eye.** Significant changes of decreased thickness were found in the central, temporal superior, nasal superior and nasal macular areas in eyes after postoperative endophthalmitis. Compared to eyes after uncomplicated phacoemulsification (221.86 μm ± 28.47) mean choroidal thickness is significantly thinner in eyes after endophthalmitis (195.14 μm ± 23.19), (p = 0.018).

**Figure 5:**
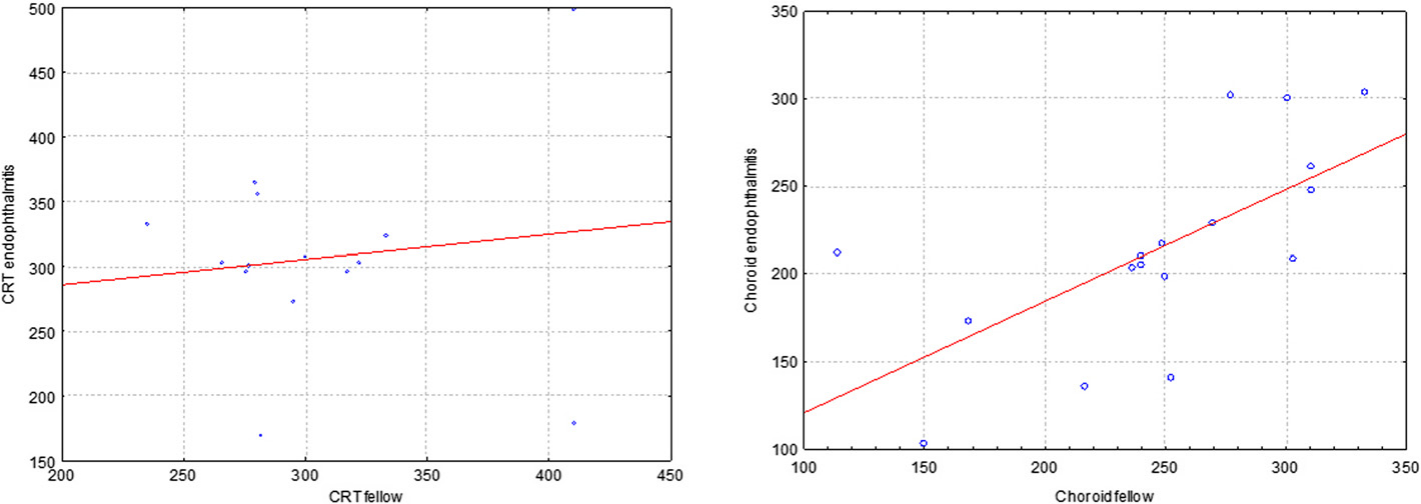
**No correlation was seen between CRT and subfoveal choroidal thickness in the study and fellow eyes.** (Spearman Rank Order Correlation, p > 0.05).

Choroidal thickness in the central, temporal superior, temporal inferior, nasal superior and nasal central region was found significantly lower in the study eyes (p = 0.03, 0.007, 0.09, 0.02 and 0.049, respectively). In other regions, choroidal thickness was also decreased, the difference was insignificant (p = 0.33, 0.36) (Figure 4). In the study eyes, mean choroidal thickness was significantly lower compared to the control eyes (195.14 ± 23.19 μm and 221.86 ± 28.47 μm, respectively, p = 0.018) (Figure 4). There was no significant correlation between central retinal thickness and choroidal thickness of the study (p = 0,136) and fellow eyes in the foveal region (p = 0.714) (Figure 5).

## Discussion

Postoperative endophthalmitis is still the most dangerous complication after cataract surgery. Former studies [2,3] presented evidence based guidelines for the treatment and management of this eye infection. For severe postoperative endophthalmitis with severe vision loss, vitrectomy seems to be the first choise of treatment [3] but the empiric treatment with broad–spectrum antibiotics is also important for successful clinical outcomes [2,17]. The goal of this study was to assess the clinical and morphological changes in the retina and choroid long time after postoperative endophthalmitis.

The introduction of spectral domain optical coherence tomography brought a series of improvements in comparison to time domain OCT [6]. In the last 3–4 years, new approaches and technical developments opened new ways in optical coherence tomography and options for examination of retinal and choroidal structures [18]. The Spectralis OCT system is one of the numerous commercially available SD-OCT instruments [11,14], being the first capable of performing enhaced depth imaging (EDI). Choroidal imaging may have possible importance as ocular and systemic disorders related to vascular changes can be associated with significant visual loss. Besides fundoscopy and angiography being the standard procedures for examining the retina in cases of presumed vascular pathologies, recent SD-OCT studies showed that vascular disorders may also cause microstructural changes in the choroid [7,19,20]. Other studies using EDI technology revealed new data about deeper structures of the optic nerve head (ONH) and the choroid [13].

In the present study involving eyes with postoperative endophthalmitis, no differences were detected in the thickness of macular retinal layers along with macular volume. Retinal thickness is one of the major treatment criteria for age-related macular degeneration or diabetic macular edema [7,12]. Apart from this, several authors reported retinal structural abnormalities in various retinal diseases, such as acute zonal occult outer retinopathy-complex diseases [21], epiretinal membranes [22], retinitis pigmentosa [23] or cone dystrophy using SD-OCT and they found significant alterations in the thickness of the outer nuclear layer (ONL). Our patients enrolled in the study reported neither diabetic macular edema nor severe aged-related macular degeneration alterations (AREDS 3 or higher classification). The investigation of ultrastructural photoreceptor abnormalities in the retina was not the goal of our study, our examinations focused on the deeper structures of the retina-choroid complex.

With regard to RNFL thickness measurements there was no significant difference between eyes after endophthalmitis and fellow eyes. Recent studies showed that SD-OCT has a high accuracy and reproducibility in ONH and RNFL measurements in glaucoma [13,14]. Patients with glaucoma were excluded from our study in order to eliminate false data of RNFL thickness due to glaucoma. According to our observations, the RNFL thickness and macular retinal thickness results were tendentially decreased compared to the study eye without reaching statistical significance. Further studies with more patients may support our results.

Since the first report of EDI-OCT, OCT imaging of the choroid has attracted the interest of clinicians and encouraged further studies of the choroid using EDI-OCT. EDI is an acquisition software option which automatically captures a high sensitive cross-selectional image of the choroid close to the “zero delay line” [6]. With increasing depth into tissue, echoes are more difficult to discern from each other. EDI technology provides an increased sensitivity of the spectrometer with a higher frequency modulation and with increased pixel number in the line scan camera. We measured choroidal thickness of the macular region in 7 points within a 20° × 20° area. Measurements were performed manually by calipers, perpendicular from the outer edge of the hyperreflective RPE to the inner sclera (choroid – sclera junction). According to histopathological examinations, the choroid measures 0.22 mm in thickness posteriorly [24]. In our study the mean choroidal thickness measurement was comparable, approximately 221.86 ± 28.47 μm. In the subfoveal region, choroidal thickness was 248.1 ± 66.2 μm in control eyes and 215.2 ± 63.4 μm after endophthalmitis, respectively. Margolis et al. and Spaide et al. reported similar measurements (mean subfoveal choroidal thickness was 287 ± 76 μm measured by the Spectralis with a sample size of 54 healthy eyes) [25]. An available software used for choroidal mapping and volume measurement (e.g. Heidelberg Eye Explorer software 5.3”) would also be appropriate to measure choroidal thickness and volume [26]; however, we did not have the opportunity to use this software for the measurements.

In the present study we found a significant thinning of choroidal thickness after endophthalmitis (p = 0.018), but there was no correlation with visual function. Furthermore, no significant differences in BCVA were observed in eyes after the healing of postoperative endophthalmitis. The patients were of older age, with a range of 56 to 89 years, one patient had amblyopia in the control eye which might have caused the large SD of our BCVA data.

So far, choroidal thickness is not widely used as a major criterion to follow up the treatment of macular or choroidal diseases. As an example, in Vogt-Koyanagi-Harada disease choroidal thickness is reduced after succesful steroid treatment; therefore, can be an important indicator for the assessment of corticosteroid treatment efficacy [16]. Recent studies also showed a decrease in choroidal thickness in highly myopic eyes [27,28] which is supposed to be a significant risk for the developement of choroidal neovascularisation. Other recently published data showed that macular choroidal thickness is not influenced by intraocular pressure [29]. It has been presumed that choroidal thickness influences the posterior eye wall thickness. Németh et al. found in ultrasound measurements that the ocular wall was thicker in hypotony and patients with exophthalmus, but ocular wall dimensions were smaller in patients with glaucoma [30]. Other measurements with scanning laser Doppler flowmeter showed a reduced retinal microcirculation in myopic and glaucomatous eyes [31]. Guthoff et al. and Németh et al. showed that in healthy persons the thickness of the ocular wall is very closely dependent on the axial length of the eye, and that the volume of the wall of the eye is nearly constant [32]. Choroidal thickness may probably not be an absolute indicator for failure or success of treatment for endophthalmitis, but decreased choroidal thickness can explain unexpected clinical outcomes with poor vision.

Our study reports the evaluation of a small case series of patients with postoperative endophthalmitis. As inclusion criteria we evaluated only severe acute postcataract endophthalmitis cases with poor inicial visual acuity. Pars plana vitrectomy was performed in each case within 24 hours after the outbreak of endophthalmitis, there were no complications observed either intraoperatively or in the early postoperative period and clear media were obtained in each case within 4 weeks. We found that retinal structure and thickness were not significantly different in both groups even long time after vitrectomy. Fujiwara et al. also showed recently that there were no changes in choroidal thickness after microincision vitrectomy for ERM and macular hole [33]. Supposing the retina is more exposed to some traumatic events during vitrectomy it may be presumed that choroidal thickness changes were probably due to decreased perfusion caused by the postcataract endophthalmitis. Thus, our findings may also support the theses that early vitrectomy may be of important benefit for long term clinical outcomes in such cases.

It should be noted that out of 17 patients only 8 specimens provided a positive microbiological culture. In other studies a different range of microorganisms was isolated from vitreus samples (70 - 90%) [34-36].

In the present study we evaluated retinal thickness, choroidal thickness and major retinal abnormalities after postcataract endophthalmitis. However, our study had some limitations. A larger, prospective series of patients and the detailed evaluation also of the mictrostructural changes in the outer retinal layers, especially in the external limiting membrane (ELM) and the continuity of the inner segment-outer segment junction (IS/OS junction) could provide more information on visual acuity changes after severe postcataract endophthalmitis. Nevertheless, a larger case series could contribute to a more sophisticated statistical evaluation such as correlation analysis with the timing of surgery, the length of follow up time or some surgical factors, such as posterior hyaloid detachment, type of pathogens and age, therefore a further prospective study is warranted.

## Conclusion

In this paper, we not only summarize a review of actual data on measurements with spectral domain OCT but also show a new application to examine morphological changes of the posterior eye wall in postcataract endophthalmitis. We found that choroidal thickness showed significant decrease in patients who underwent pars plana vitrectomy due to acute postoperative endophthalmitis after cataract surgery. The results of this study indicate that severe acute endophthalmitis leads to thickness changes in the choroid and we presume that endophthalmitis could cause some changes alteration in its perfusion system. Increased macular retinal thickness and development of epiretinal membranes may be associated with performed vitrectomy or endophthalmitis itself. The absence of other significant structural and morphological findings of the retina shows that successful treatment may guarantee satisfactory long-term clinical results even long after this severe postoperative complication. OCT and EDI-OCT is an easy, reproducible [37] and noninvasive examination while providing a better understanding of ocular infections and their morphological changes.

## Competing interests

The authors declare that they have no competing interests' or relationship with any organization that produces any devices used in the study.

## Authors’ contributions

OM recruited the patients, wrote the manuscript, participated in study design, ethical approval. ÉV carried out the measurements. GM and IS helped in formatting, language, reviewed the literature. ZG participated in study design, critical reading of the manuscript, JN provided equipments and facility, study design. MR organized ethical approval, performed the statistical analysis and helped to draft the manuscript. All authors read and approved the final manuscript.

## Authors’ information

OM is an ophthalmologist with his main field of interests including medical retina, intraocular infections and pediatric ophthalmology. A part of this work has been presented as poster at the DOG 2012 (Berlin, Germany, September 2012) and awarded with the DOG Travel Award 2012.

## Pre-publication history

The pre-publication history for this paper can be accessed here:

http://www.biomedcentral.com/1471-2415/14/76/prepub
